# Supplementary material for: Distinct information conveyed to the olfactory bulb by feedforward input from the nose and feedback from the cortex
Source: Nat Commun. 2024 Apr 16;15:3268. doi: 10.1038/s41467-024-47366-6 (PMC11021479; doi:10.1038/s41467-024-47366-6)
Supplement: Supplementary file 1 — Supplementary Information [file 41467_2024_47366_MOESM1_ESM.pdf]

Distinct information conveyed to the olfactory bulb by feedforward input from the nose and feedback from the cortex

Zak et al.

Supplementary Figure 1

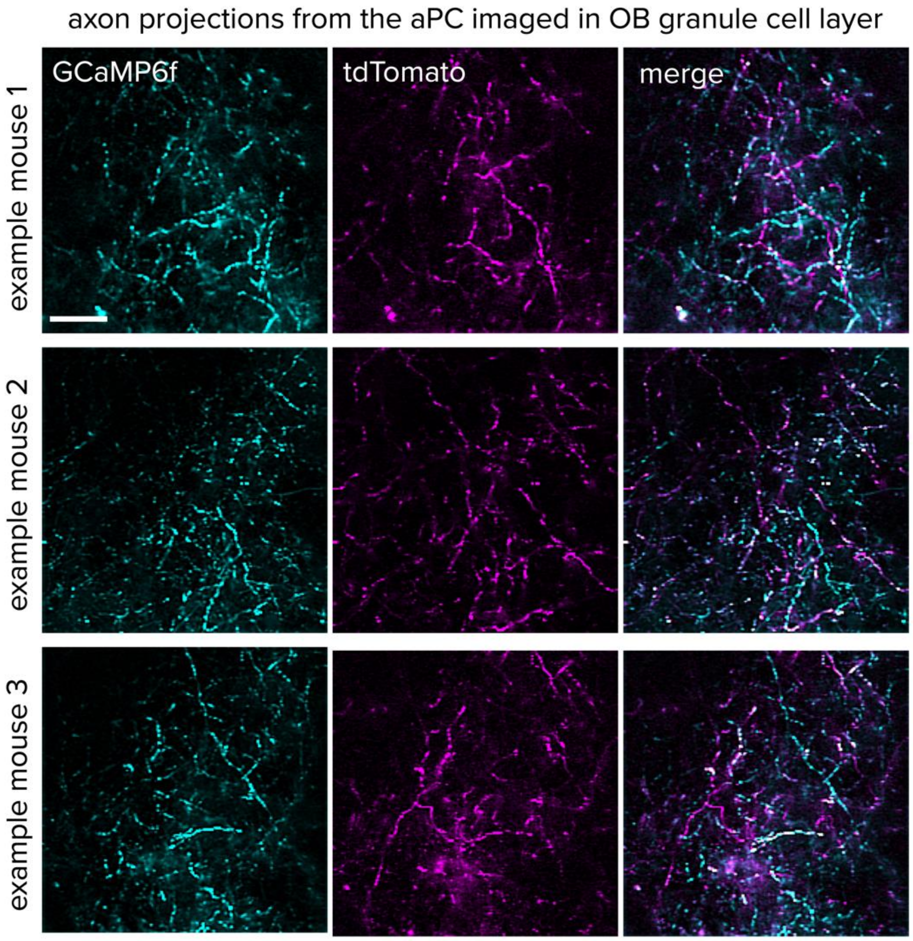

Co-labeled cortical axons in the olfactory bulb.
Example imaging fields through a cranial window from three live mice. Cortical projections to the OB are labeled with GCaMP6f (left) and tdTomato (middle). Merged image at the right. scale bar = 20 µm. Images are representative of three individual imaging fields.

Supplementary Figure 2

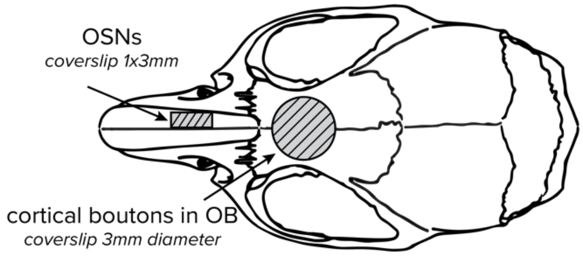

Imaging locations for OSNs and cortical boutons in the OB
A bone-thinning procedure is used to gain optical access to the dorsal recess of the olfactory epithelium (see Methods). A 3 mm diameter coverslip is implanted into a craniotomy over both olfactory bulbs.

Supplementary Figure 3

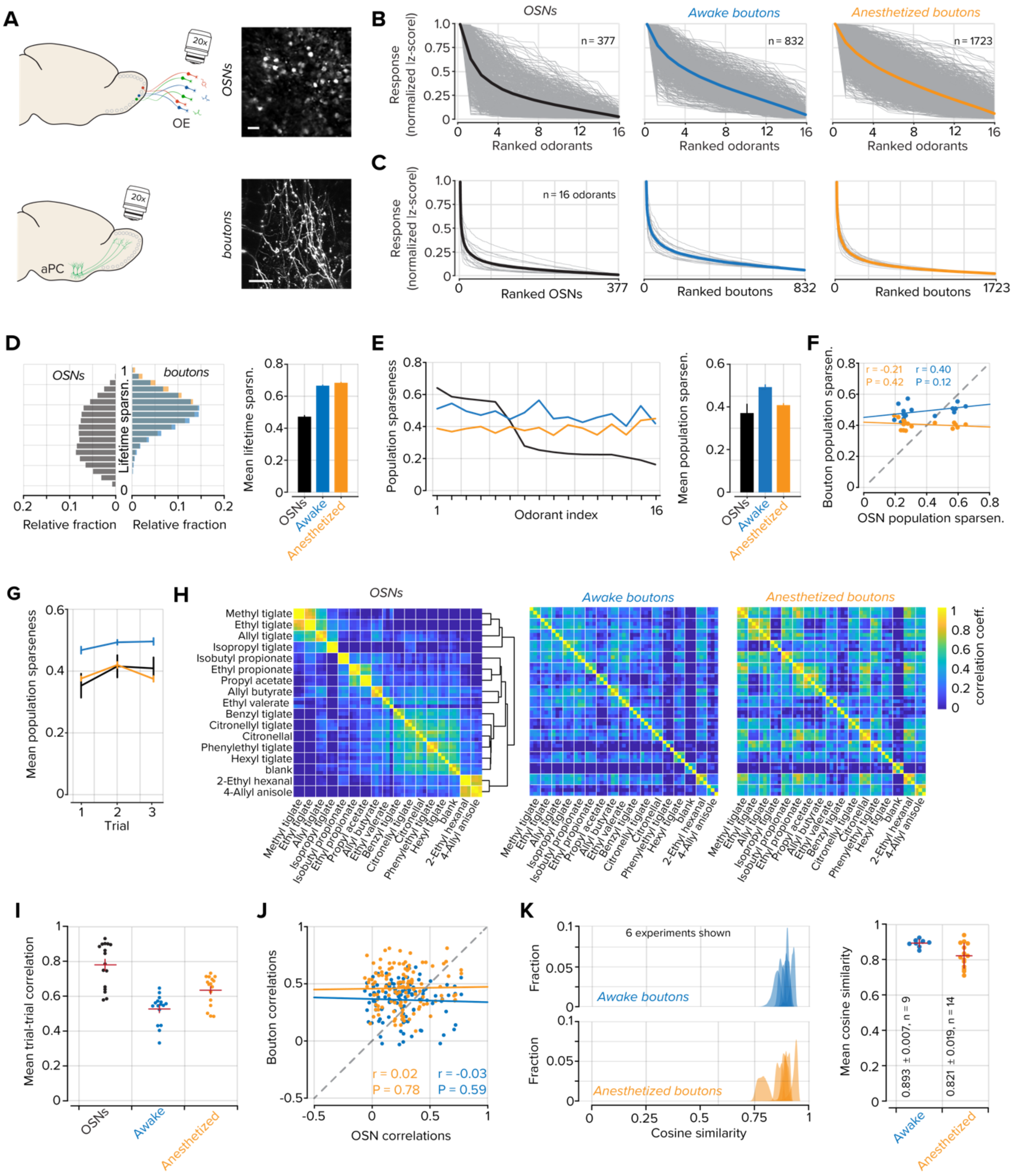

Odorant tuning in awake and anesthetized boutons.
A. Top, example images of GCaMP3 expressing OSNs in the olfactory epithelium, scale bar = 20 μm. Bottom, GCaMP6f expressing cortical projections to the OB, scale bar = 20 μm. **B**. Normalized and ranked responses to 16 odorants in OSNs (black; n = 377), cortical projections in awake mice (blue, n = 832), and in anesthetized mice (orange; n = 1723). Each tuning curve is independently sorted and ranked. Gray lines represent individual ROIs, and thick-colored lines represent the mean of all ROIs. **C**. Normalized and ranked responses of each OSN, cortical projection in awake mice, and anesthetized mice for each of the 16 odorants. Gray lines represent individual odorants, and thick-colored lines represent the mean of all odorants. **D**. Left, Distributions of lifetime sparseness measured in OSNs (black), cortical projections in awake mice (blue), and anesthetized mice (orange). Right, mean lifetime sparseness was measured in OSNs (black; 0.24 ± 0.01), cortical projections in awake mice (blue; 0.36 ± 0.01), and anesthetized mice (orange; 0.47 ± 0.01). $P < 0.001$, all comparisons; Kolmogorov-Smirnov test. Error bars represent s.e.m. **E**. Left, population sparseness for each of 16 odorants sorted to OSN values. Right, mean population sparseness for all odorants (0.08 ± 0.02 OSNs, 0.02 ± 0.01 awake boutons, 0.11 ± 0.01 anesthetized boutons; $P < 0.001$; Kruskal-Wallis test). Error bars represent s.e.m. **F**. Scatter plot of the relationship between OSN population sparseness and bouton population sparseness in awake or anesthetized mice (anesthetized boutons to OSNs r = 0.27; P = 0.31; chi-squared test; awake boutons to OSNs r = 0.13; P = 0.63; chi-squared test). **G**. Mean population sparseness for each of three trials. Error bars represent s.e.m. **H**. Odorant-odorant correlations in OSNs, awake boutons, and anesthetized boutons. Individual odorants are bounded by white lines and each odorant contains three trials. Hierarchical clustering was used to group similar odorants in OSNs and the clusters were then used to group datasets in boutons. **I**. Variability within trials of the same odorants in OSNs and awake or anesthetized boutons. $P < 0.001$ Kruskal-Wallis test. The horizontal red bar denotes the mean and the vertical red bars represent s.e.m. **J**. Scatter plot of the relationship between odorant-odorant correlations in OSNs and awake or anesthetized boutons (awake boutons to OSNs r = -0.03; P = 0.59; chi-squared test; anesthetized boutons to OSNs r = 0.02; P = 0.78; chi-squared test). **K**. Decreased correlations in awake boutons are not the product of motion artifacts. Left, distributions of the cosine similarity between a template image and each frame in an imaging session from six example experiments in awake and anesthetized preparations. Right, summary data of all imaging sessions. The horizontal red bar denotes the mean and the vertical red bars represent s.e.m. n = 9 awake fields and 14 anesthetized fields. P = 0.145; Rank-sum test. The underlying data for each plot are available in the source data file.

Supplementary Figure 4

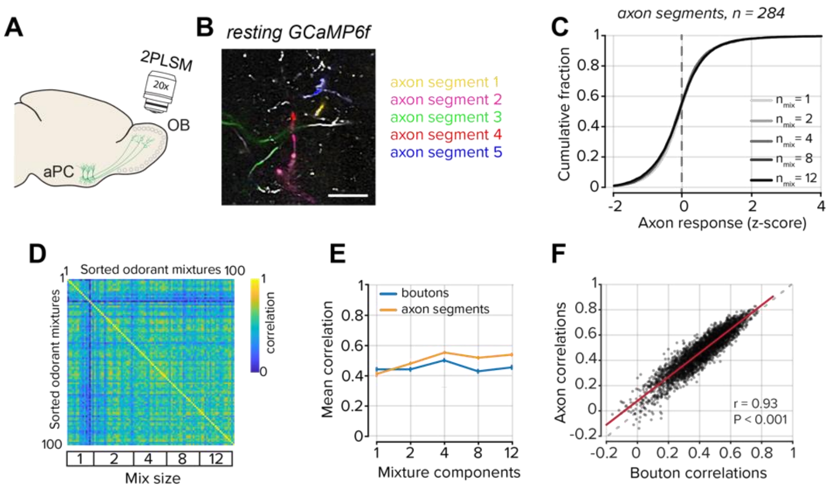

Odorant mixture correlations in axon segments.
A. Cortical axons expressing GCaMP6f were imaged in the OB through cranial windows. **B**. GCaMP6f expressing cortical projections to the OB. Identified axon segments are labeled in colors. scale bar = 20 μm. **C**. Distributions of axon segment responses to odorant mixtures of increasing size. Color shade corresponds to the mixture size. **D**. Correlation matrix of mixture-mixture relationships in axon segments. **E**. Plot of the mean correlation of all mixtures at each mixture complexity, axon segments in orange, boutons from **Figure 5** in blue. Error bars represent s.e.m. **F**. Scatter plot of the relationship between mixture-mixture correlations measured in axon segments and boutons ($r = 0.93$; $P < 0.001$; chi-squared test). The underlying data for each plot are available in the source data file.

Supplementary Figure 5

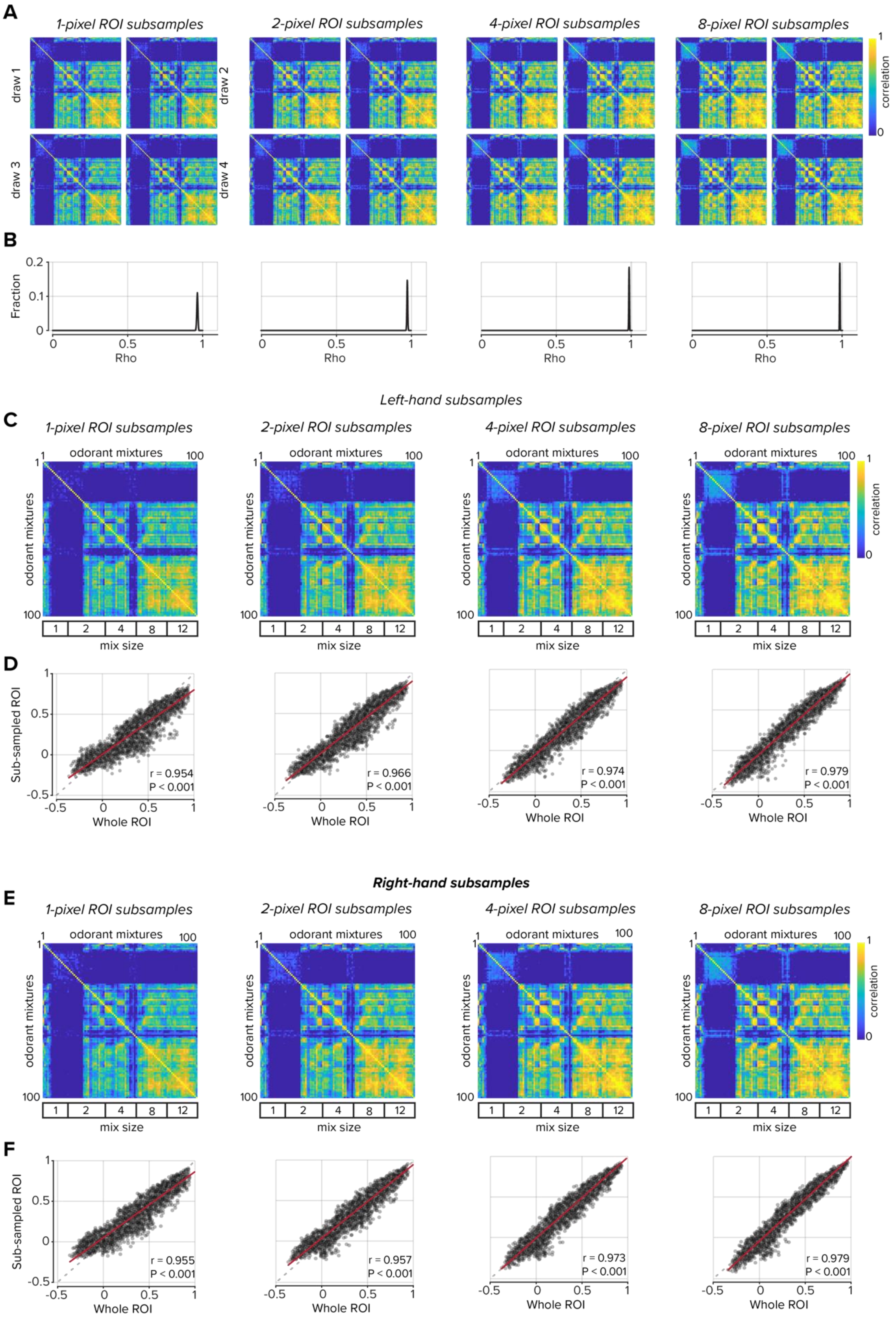

Odorant mixture correlations in subsampled OSN regions of interest.

A. Each OSN's ROI was subsampled to randomly select one, two, four, or eight pixels. Mixture-mixture correlations were then computed as in **Figure 6A**. Four example draws are shown for each subsample. **B**. Each subsample size was drawn 500 times and the mixture-mixture correlations of each subsample were then compared to the whole-ROI data (**Figure 6A**). Distributions of rho values from linear regressions are plotted. For each regression, $P < 0.001$; chi-squared test. A single pixel from each OSN was sufficient to reproduce the whole-ROI correlation matrix. **C**. A second subsampling approach was used to select the one, two, four, or eight most pixels at the whole-ROI left-hand boundary. Mixture-mixture correlations were then computed. **D**. Comparison of the subsamples of each size and the whole-ROI mixture-mixture correlations. **E-F**. Same as parts C-D using left-hand subsamples. The underlying data for each plot are available in the data repository indicated in the Data Availability statement.

Supplementary Figure 6

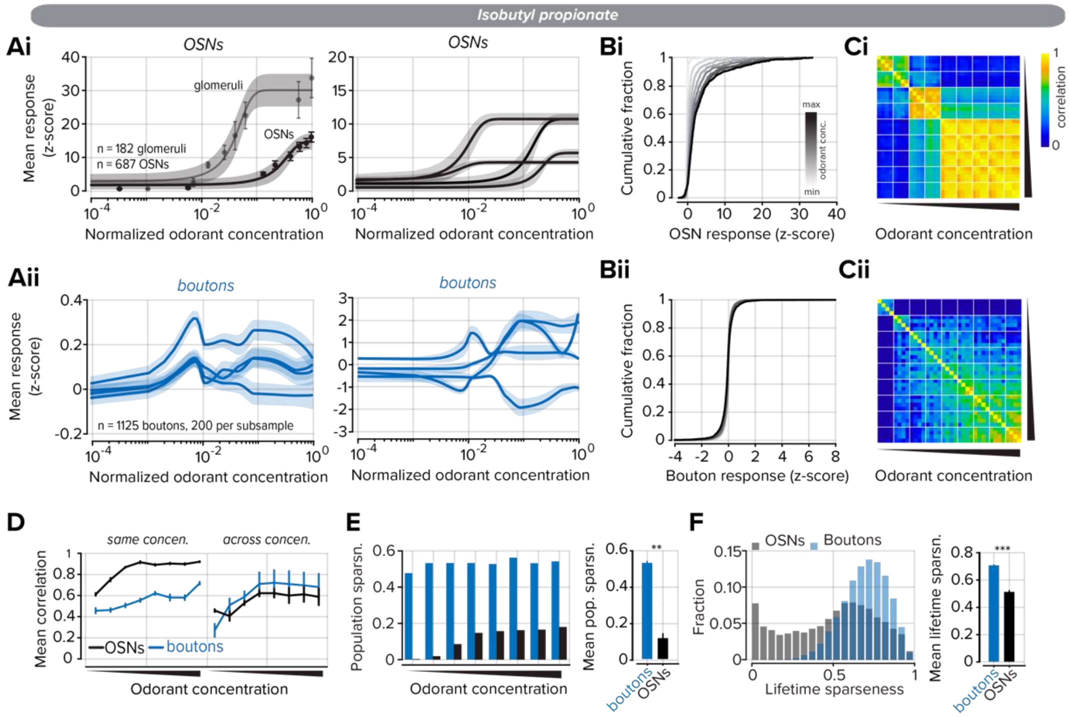

Nonmonotonic concentration responses in cortical boutons in response to the odorant Isobutyl propionate.

Ai. *Left*, odorant concentration responses were measured in OSN somata in the OE and their axon terminals within the glomerular layer. Errors are expressed as s.e.m. on individual data points. The solid line denotes a sigmoidal fit to the individual data points, and the shaded area denotes the 95% confidence interval for the sigmoidal fit. *Right*, four example OSN responses with the sigmoidal fit (solid) and 95% confidence interval (shaded) **Aii**. *Left*, odorant concentration responses were measured in cortical boutons in the OB. Four traces show subsamples selected from 200 boutons each. Data were fitted with an Akima piecewise cubic Hermite interpolation (solid line). The shaded area represents the 95% confidence interval. *Right*, four examples of individual bouton responses. **B**. Distributions of OSN (**Bi**) and bouton (**Bii**) responses to increasing odorant concentration. Color shade corresponds to the odorant concentration. **C**. Correlation matrix of odorant responses to increasing odorant concentration in OSNs (**Ci**) and boutons (**Cii**). White lines bound concentrations, and each concentration contains four trials. **D**. Left, OSN, and bouton correlations between the same odorant concentration trials. Right, OSN and bouton correlations across concentrations. **E**. Left, population sparseness at each odorant concentration for boutons (blue, n = 8 concentrations) and OSNs (black, n = 8 concentrations). Right, summary data of mean population sparseness for all concentrations. Sign rank test, error bars represent s.e.m. **F**. Left, Distributions of lifetime sparseness measured at all concentrations for boutons (blue, n =1125) and OSNs (black, n = 687). Right, summary data of mean lifetime sparseness. Kolmogorov-Smirnov test, error bars represent s.e.m. ** denotes $P < 0.01$, *** denotes $P < 0.001$. The underlying data for each plot are available in the source data file.

Supplementary Figure 7

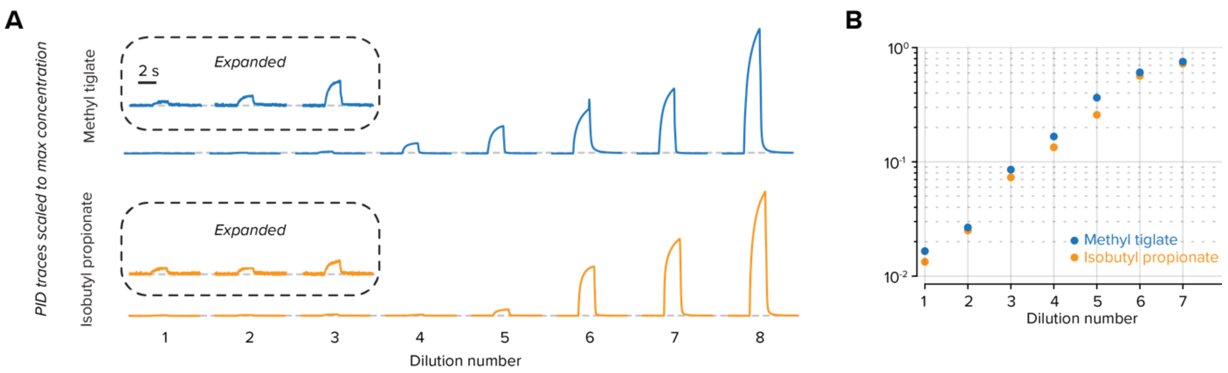

Photoionization detector (PID) traces for odorant concentrations.
A. PID traces for concentrations of Methyl tiglate (**Figure 6**) and Isobutyl propionate (**Supplemental Figure 5**) at eight v/v dilutions (see Methods). Odorants were delivered for 2 s. Expanded traces show PID signals at the lowest concentrations. **B**. For each v/v dilution, the area under the PID trace was measured and plotted relative to the highest concentration.

Supplementary Figure 8

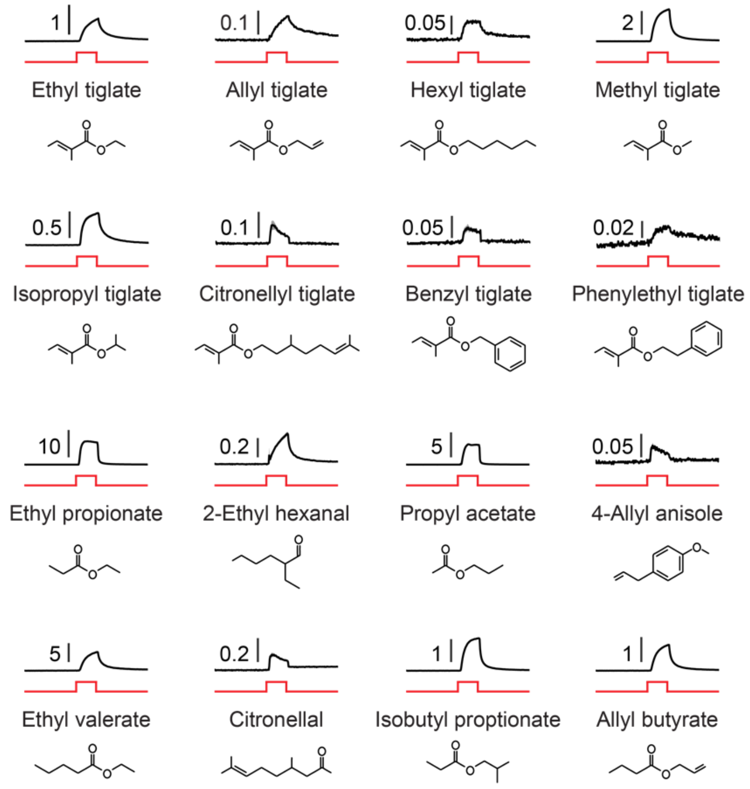

Photoionization detector (PID) traces for odorants in Supplementary Table 1
PID traces of all odorants used in tuning and mixture experiments in black (mean of 5 trials), voltage command to olfactometer in red. The molecular shape of each odorant is below the corresponding traces. Scale bar units are volts.

Supplementary Table 1

Index	Name/Formula	Structure	CAS number	Molecular Weight (g/mol)	Standard Vapor Pressure (mmHg at 25°C)
1	Ethyl tiglate $C_7H_{12}O_2$	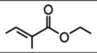	5837-78-5	128.17	4.2690
2	Allyl tiglate $C_8H_{12}O_2$	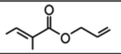	7493-71-2	140.18	1.2720
3	Hexyl tiglate $C_{11}H_{20}O_2$	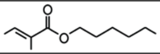	16930-96-4	184.27	0.0520
4	Methyl tiglate $C_6H_{10}O_2$	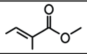	6622-76-0	114.14	13.3790
5	Isopropyl tiglate $C_8H_{14}O_2$	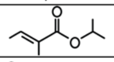	1733-25-1	142.20	1.8770
6	Citronellyl tiglate $C_{15}H_{26}O_2$	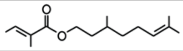	24717-85-9	238.37	0.0036
7	Benzyl tiglate $C_{12}H_{14}O_2$	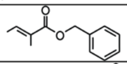	37526-88-8	190.24	0.0010
8	Phenylethyl tiglate $C_{13}H_{16}O_2$	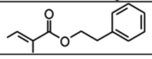	55719-85-2	204.27	0.0010
9	Ethyl propionate $C_5H_{10}O_2$	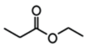	105-37-3	102.13	35.9000
10	2-Ethyl hexanal $C_8H_{16}O$	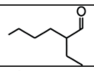	123-05-7	128.21	1.8000
11	Propyl acetate $C_5H_{10}O_2$	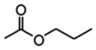	109-60-4	102.13	35.2230
12	4-Allyl anisole $C_{10}H_{12}O$	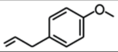	140-67-0	148.20	0.1650
13	Ethyl Valerate $C_7H_{12}O_2$	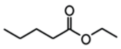	539-82-2	130.18	4.7450
14	Citronellal $C_{10}H_{18}O$	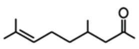	106-23-0	154.25	0.2800
15	Isobutyl propionate $C_7H_{14}O_2$	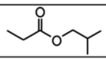	540-42-1	130.18	6.4700
16	Allyl butyrate $C_7H_{12}O_2$	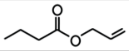	2051-78-7	128.17	4.4430

Odorant information

Properties of 16 monomolecular odorants used in this study.

Supplementary Table 2

	Effective odorants	Lifetime sparseness	Population sparseness	Intertrial correlation
**OSNs**	3.15 ± 0.15	0.46 ± 0.01	0.37 ± 0.04	0.78 ± 0.03
**Boutons**	4.45 ± 0.13	0.65 ± 0.01	0.49 ± 0.01	0.53 ± 0.02
**P-value**	< 0.001	< 0.001	0.013	< 0.001
**Statistical Test**	Rank-sum	Kolmogorov-Smirnov	Sign-rank	Sign-rank

Data related to Main Figure 3

Supplementary Table 3

mixture size	mixture component indices	mixture size	mixture component indices
2	13,15	8	2,5,6,7,10,12,14,15
2	5,12	8	1,2,6,7,8,10,11,14
2	4,8	8	1,4,5,9,10,11,12,15
2	1,15	8	1,4,5,6,9,10,11,16
2	1,12	8	1,6,10,11,12,13,14,16
2	2,3	8	1,4,6,8,9,10,12,14
2	9,11	8	1,2,3,4,7,9,11,15
2	1,4	8	5,6,7,8,10,11,14,16
2	6,9	8	1,2,3,5,6,8,11,15
2	4,13	8	1,6,8,10,12,13,14,16
2	3,4	8	1,3,4,5,9,10,12,16
2	13,14	8	2,3,6,7,9,12,13,15
2	9,15	8	1,4,9,10,11,14,15,16
2	3,11	8	1,4,5,8,10,11,12,14
2	7,12	8	3,5,6,9,10,13,15,16
2	5,15	8	1,2,3,4,9,11,13,16
2	8,15	8	2,3,4,7,8,10,11,15
2	12,14	8	2,3,4,5,6,12,14,16
2	2,6	8	1,4,5,7,12,14,15,16
2	1,7	8	2,3,5,6,9,10,11,16
2	10,16	12	2,4,7,8,9,10,11,12,13,14,15,16
2	3,12	12	2,3,4,6,7,8,9,10,11,12,14,16
2	1,16	12	1,3,4,5,6,7,9,10,11,13,14,15
2	4,11	12	1,3,4,6,7,8,9,10,12,13,15,16
4	3,4,10,11	12	1,2,3,4,6,8,9,10,12,14,15,16
4	1,2,4,6	12	1,2,3,5,8,9,11,12,13,14,15,16
4	4,5,6,14	12	1,2,3,4,6,9,11,12,13,14,15,16
4	4,6,10,13	12	1,3,4,5,6,7,8,10,11,12,13,15
4	6,13,14,15	12	1,4,5,6,7,9,11,12,13,14,15,16
4	1,5,6,8	12	2,3,5,6,7,8,9,10,11,12,13,16
4	3,7,10,14	12	1,2,3,4,6,7,8,9,11,12,14,15
4	9,10,11,12	12	1,2,3,4,5,7,8,9,11,12,14,15
4	2,3,4,14	12	1,2,3,4,5,6,7,8,11,12,13,15
4	3,10,14,15	12	3,4,6,7,9,10,11,12,13,14,15,16
4	2,8,15,16	12	1,3,5,6,7,9,10,11,12,13,14,15
4	7,8,11,12	12	1,2,3,5,6,8,10,12,13,14,15,16
4	2,4,12,14	12	1,2,5,6,7,8,9,10,12,14,15,16
4	5,6,9,11	12	2,3,4,5,7,8,10,11,12,13,15,16
4	9,11,12,16	12	3,4,6,7,8,9,10,11,12,13,14,15
4	1,2,3,12	12	3,5,6,7,8,9,11,12,13,14,15,16
4	8,12,14,15		
4	3,4,8,9		
4	2,9,10,11		
4	3,8,9,13		

Odorant mixture compositions

Odorant mixture compositions Related to Figures 4 & 5. Odorant component indices are consistent with Supplementary Table 1.

Supplementary Table 4

	Mixture Size	1	2	4	8	12
**Mean Z-Score**	OSNs	0.345 ± 0.007	0.474 ± 0.007	0.651 ± 0.009	0.899 ± 0.011	0.948 ± 0.011
	Boutons	0.334 ± 0.003	0.357 ± 0.003	0.344 ± 0.003	0.382 ± 0.003	0.368 ± 0.003
	P-value	< 0.001	< 0.001	< 0.001	< 0.001	< 0.001
	Statistical Test	Rank-sum	Rank-sum	Rank-sum	Rank-sum	Rank-sum
**Lifetime sparseness**	OSNs	0.511 ± 0.007	0.528 ± 0.005	0.622 ± 0.005	0.703 ± 0.005	0.755 ± 0.005
	Boutons	0.645 ± 0.004	0.659 ± 0.004	0.669 ± 0.004	0.663 ± 0.004	0.654 ± 0.004
	P-value	< 0.001	< 0.001	< 0.001	< 0.001	< 0.001
	Statistical Test	Rank-sum	Rank-sum	Rank-sum	Rank-sum	Rank-sum
**Population sparseness**	OSNs	0.483 ± 0.026	0.457 ± 0.019	0.401 ± 0.018	0.412 ± 0.016	0.449 ± 0.012
	Boutons	0.461 ± 0.014	0.472 ± 0.009	0.474 ± 0.012	0.481 ± 0.010	0.479 ± 0.013
	P-value	0.200	0.819	0.002	0.002	0.117
	Statistical Test	Sign-rank	Sign-rank	Sign-rank	Sign-rank	Sign-rank

Data related to Main Figure 4

Supplementary Table 5

	Inter-concentration correlation	Across concentration correlation	Population sparseness	Lifetime sparseness
**OSNs**	0.771 ± 0.026	0.518 ± 0.404	0.504 ± 0.032	0.508 ± 0.007
**Boutons**	0.546 ± 0.026	0.503 ± 0.402	0.180 ± 0.050	0.686 ± 0.004
**P-value**	0.005	0.945	0.008	< 0.001
**Statistical Test**	Rank-sum	Sign-rank	Sign-rank	Kolmogorov-Smirnov

Data related to Main Figure 6
