# Supplementary material for: Distinct information conveyed to the olfactory bulb by feedforward input from the nose and feedback from the cortex
Source: Nat Commun. 2024 Apr 16;15:3268. doi: 10.1038/s41467-024-47366-6 (PMC11021479; doi:10.1038/s41467-024-47366-6)
Supplement: Supplementary file 3 — Reporting Summary [file 41467_2024_47366_MOESM3_ESM.pdf]

nature portfolio

Corresponding author(s): Joseph D. Zak

Last updated by author(s): Feb 22, 2024

Reporting Summary

Nature Portfolio wishes to improve the reproducibility of the work that we publish. This form provides structure for consistency and transparency in reporting. For further information on Nature Portfolio policies, see our Editorial Policies and the Editorial Policy Checklist.

Statistics

For all statistical analyses, confirm that the following items are present in the figure legend, table legend, main text, or Methods section.

n/a	Confirmed	
☐	☒	The exact sample size (*n*) for each experimental group/condition, given as a discrete number and unit of measurement
☐	☒	A statement on whether measurements were taken from distinct samples or whether the same sample was measured repeatedly
☐	☒	The statistical test(s) used AND whether they are one- or two-sided<br>*Only common tests should be described solely by name; describe more complex techniques in the Methods section.*
☒	☐	A description of all covariates tested
☐	☒	A description of any assumptions or corrections, such as tests of normality and adjustment for multiple comparisons
☐	☒	A full description of the statistical parameters including central tendency (e.g. means) or other basic estimates (e.g. regression coefficient) AND variation (e.g. standard deviation) or associated estimates of uncertainty (e.g. confidence intervals)
☐	☒	For null hypothesis testing, the test statistic (e.g. *F*, *t*, *r*) with confidence intervals, effect sizes, degrees of freedom and *P* value noted<br>*Give P values as exact values whenever suitable.*
☒	☐	For Bayesian analysis, information on the choice of priors and Markov chain Monte Carlo settings
☒	☐	For hierarchical and complex designs, identification of the appropriate level for tests and full reporting of outcomes
☐	☒	Estimates of effect sizes (e.g. Cohen's *d*, Pearson's *r*), indicating how they were calculated

Our web collection on statistics for biologists contains articles on many of the points above.

Software and code

Policy information about availability of computer code

Data collection	Data were collected using LabVIew (2016) to control a multiphoton scansystem and filewriting
Data analysis	maging data were processed using freely available packages including NoRmCorre v0.1.1 (https://github.com/flatironinstitute/NoRMCorre) and Caiman vl.4.1 (https://github.com/flatironinstitute/CalmAn). All analysis was performed using custom code written in Matlab2022a. Analysis code is available at https://github.com/JDZak-Lab/Zak-et-al.-2024-Nat.-Comm.

For manuscripts utilizing custom algorithms or software that are central to the research but not yet described in published literature, software must be made available to editors and reviewers. We strongly encourage code deposition in a community repository (e.g. GitHub). See the Nature Portfolio guidelines for submitting code & software for further information.

Data

Policy information about availability of data

All manuscripts must include a data availability statement. This statement should provide the following information, where applicable:

- Accession codes, unique identifiers, or web links for publicly available datasets
- A description of any restrictions on data availability
- For clinical datasets or third party data, please ensure that the statement adheres to our policy

The data supporting this study’s findings have been deposited in a Github data base under the accension code xxx (https://github.com/JDZak-Lab/Zak-et-al.-2024-Nat.-Comm.) Source data are provided with this paper.

Research involving human participants, their data, or biological material

Policy information about studies with human participants or human data. See also policy information about sex, gender (identity/presentation), and sexual orientation and race, ethnicity and racism.

Reporting on sex and gender	N/A
Reporting on race, ethnicity, or other socially relevant groupings	N/A
Population characteristics	N/A
Recruitment	N/A
Ethics oversight	N/A

Note that full information on the approval of the study protocol must also be provided in the manuscript.

Field-specific reporting

Please select the one below that is the best fit for your research. If you are not sure, read the appropriate sections before making your selection.

☒ Life sciences ☐ Behavioural & social sciences ☐ Ecological, evolutionary & environmental sciences

For a reference copy of the document with all sections, see nature.com/documents/nr-reporting-summary-flat.pdf

Life sciences study design

All studies must disclose on these points even when the disclosure is negative.

Sample size	Our experiments were designed to use 5-6 mice in each experimental group. The final numbers of mice were affected by surgical and imaging success rate. The number of mice was chosen so that statistics on imaging fields could be performed. From each animal multiple imaging fields can be used. Both boutons and individual sensory neurons were collected from the most optically dense imaging area in each animal. In some animals multiple imaging fields were used. Our sample size is consistent with studies in the field.
Data exclusions	Mice with unsuccessful surgeries or poor imaging quality are excluded.
Replication	All experiments were at least performed in three or more mice for the reproducibility. Replication was successful and consistent across animals.
Randomization	All animals were of the same genotype in this study.
Blinding	Blinding was not necessary because there were no group comparisons in this study.

Reporting for specific materials, systems and methods

We require information from authors about some types of materials, experimental systems and methods used in many studies. Here, indicate whether each material, system or method listed is relevant to your study. If you are not sure if a list item applies to your research, read the appropriate section before selecting a response.

Materials & experimental systems

n/a	Involved in the study	
☒	☐	Antibodies
☒	☐	Eukaryotic cell lines
☒	☐	Palaeontology and archaeology
☐	☒	Animals and other organisms
☒	☐	Clinical data
☒	☐	Dual use research of concern
☒	☐	Plants

Methods

n/a	Involved in the study	
☒	☐	ChIP-seq
☒	☐	Flow cytometry
☒	☐	MRI-based neuroimaging

April 2023

Animals and other research organisms

Policy information about studies involving animals; ARRIVE guidelines recommended for reporting animal research, and Sex and Gender in Research

Laboratory animals	Mice were acquired from an in house breeding stock of OMP-GCaMP3 animals (Isogai et al., 2011) or C57B16. All surgeries and experiments were carried out in adult mice (8 weeks or older) of both sexes.
Wild animals	This study did not involve wild animals.
Reporting on sex	Animals of both sexes were used in this study. A roughly equal number of each sex was used where possible. Sex was not considered as a biological variable study and is not relevant to our outcomes.
Field-collected samples	This study did not involve samples collected from the field.
Ethics oversight	All the experiments were performed in accordance with the guidelines set by the National Institutes of Health and approved by the Institutional Animal Care and Use Committee at Harvard University (protocol 29-20) or the University of Illinois Chicago (protocol 22-011).

Note that full information on the approval of the study protocol must also be provided in the manuscript.

Plants

Seed stocks	N/A
Novel plant genotypes	N/A
Authentication	N/A
